# Daytime Sleepiness in Children With Asthma: Examining Respiratory and Non-respiratory Factors

**DOI:** 10.7759/cureus.40370

**Published:** 2023-06-13

**Authors:** Abigail R Strang, Lauren Covington, Seema Rani, David Gao, Micayla Flores, Kimberly Canter, Freda Patterson, Aaron Chidekel

**Affiliations:** 1 Pediatric Pulmonology, Nemours Children's Health System, Wilmington, USA; 2 School of Nursing, University of Delaware, Newark, USA; 3 Center for Healthcare Delivery Science, Nemours Children's Health System, Wilmington, USA; 4 Sleep and Health Research Program, Department of Behavioral Health and Nutrition, College of Health Sciences, University of Delaware, Newark, USA

**Keywords:** daytime function, quality of life, sleep health, daytime sleepiness, pediatric asthma

## Abstract

Objective

Daytime sleepiness is common in youth with asthma (YWA). Treatments designed to mitigate daytime sleepiness in YWA require an understanding of the primary causes of this problem. We examined respiratory- and non-respiratory-related factors associated with daytime sleepiness in YWA.

Methods

One hundred YWA (eight to 17 years old) were included in a cross-sectional study. Daytime sleepiness, quality of life, anxiety, bedtime cellphone use, and respiratory symptoms were self-reported. Asthma severity, lung function, and the number of prescribed medications were obtained from electronic medical records. Multivariable regression models identifying variables associated with daytime sleepiness were generated.

Results

Participants were 54% male and 45% Black, with a mean age of 12.1 years. The multivariable regression model showed decreased quality of life (b = -0.328, p = 0.004) and increased bedtime cellphone use (b = 0.300, p = 0.004)were significantly related to daytime sleepiness, while anxiety (b = 0.213, p = 0.05), prescribed asthma medications (b = 0.173, p = 0.05), and worse lung function (b = -0.173, p = 0.05)were marginally related to daytime sleepiness.

Conclusions

In addition to optimizing asthma control, strategies targeting anxiety, quality of life, and nocturnal cellphone use are important to mitigate daytime sleepiness in YWA.

## Introduction

Asthma is the most common chronic medical condition in American children, affecting up to 10% of children in the United States [1]. Daytime sleepiness is common in youth with asthma (YWA) and contributes to diminished daytime function [2-4]. There are several factors associated with daytime sleepiness in YWA, including impaired sleep hygiene and bedtime technology use [5], comorbid mood disorders [6,7], asthma-related symptoms [8-11], and concurrent sleep-related airway disorders [12].

While YWA are at increased risk for sleep-related airway disorders such as obstructive sleep apnea, the prevalence of these disorders is around 23.9% [13], indicating that most YWA do not experience a primary sleep-related breathing disorder. Therefore, a comprehensive assessment of other variables (both respiratory and non-respiratory) relating to daytime sleepiness in YWA is needed in order to inform interventional studies to improve sleep health in this population.

Beyond sleep-related airway disorders, there are several plausible respiratory and non-respiratory factors that may explain the high levels of daytime sleepiness in YWA. From a respiratory perspective, asthma-related symptoms (i.e., nocturnal cough and wheeze) are common in children with chronic asthma and have been associated with increased sleep disruption (i.e., nocturnal awakenings, decreased sleep time) and subsequent daytime sleepiness [8-11]. Non-respiratory factors that may be associated with increased sleepiness in this population include poor sleep health [14], bedtime technology use and impaired sleep hygiene [5], and comorbid mood disorders [6,7]. Pediatric sleep encompasses multiple domains, including subjective satisfaction with sleep, optimal sleep timing and duration for age, sleep efficiency, daytime alertness, and positive sleep behaviors [15]. In YWA, various factors contribute to diminished sleep health and daytime sleepiness.

While there is evidence to support a range of respiratory and non-respiratory factors associated with daytime sleepiness in YWA, the relative contributions of these variables are not yet known. Specifically, to advance our understanding of daytime sleepiness in YWA and develop effective treatment strategies to mitigate this concern, it is important to identify which of the respiratory and non-respiratory factors are the key drivers of daytime sleepiness.

The primary aim of the current study is to examine the relative contributions of respiratory- and non-respiratory-related factors associated with daytime sleepiness in a sample of YWA. This article was previously presented as a meeting abstract at the Associated Professional Sleep Societies (APSS) annual meeting on June 8, 2022.

## Materials and methods

Recruitment and screening

This study was conducted at Nemours Children’s Hospital, Delaware, in Wilmington, Delaware, USA. Approval for this study was obtained from our institutional review board. Youth aged eight to 17 years with asthma were recruited to participate in the study while in the outpatient pulmonary clinic for routine visits during times of asthma control (not during exacerbations). The participants were approached by a member of the research team during a routine office visit for asthma between 2018 and 2021. Participants were eligible if they were between the ages of eight and 17 years, had a primary diagnosis of asthma, and were English-speaking. Exclusion criteria included the presence of a developmental delay that impaired the ability to independently complete the surveys and complete lung function testing and also having a primary language that was not English, as not all tools were available in languages other than English. Eligible and consenting participants completed study assessments after their routine clinical office visit.

Study measures

The dependent variable of interest is daytime sleepiness, and it was assessed using the eight-item self-reported Pediatric Daytime Sleepiness Scale, with higher scores indicating increased daytime sleepiness [16].

The independent variables were classified as being respiratory- or non-respiratory-related. Respiratory-related variables included lung function measured by spirometry testing (percent of forced expiratory volume in the first second (FEV1) of expiration predicted based on standardized values) [17], number of asthma medications prescribed, and asthma severity classification (intermittent, mild, moderate, and severe) diagnosed by the pulmonologist at the time of the visit based on published guidelines [18]. FEV1 was used as a measure of asthma control as patients routinely have spirometry performed in the pulmonology office, and a decrease in this value indicates airway obstruction. These variables were extracted from the electronic medical record at the time of the study visit. If lung function testing was not clinically necessary at the time of the study visit, the most recent baseline value within six months was used. Additional respiratory-related variables included the frequency of nocturnal respiratory symptoms (cough and wheeze) in the prior seven days and the usage of nocturnal respiratory medications, all of which were assessed using an investigator-designed self-report survey (see appendix for the investigator-designed survey).

Non-respiratory-related variables included health-related quality of life, assessed using the eight-item self-reported physical functioning subscale of the Pediatric Quality of Life Inventory (PEDS-QL) [19], and anxiety measured using an eight-item self-reported patient-reported outcomes measurement information system (PROMIS) pediatric anxiety survey short form 8a [20]. The physical functioning subscale of the PEDS-QL and PROMIS scores were converted to standardized scores based on age. Additional non-respiratory-related variables included the frequency of bedtime cellphone usage, which was assessed using an investigator-designed self-report survey (see appendix).

Study covariates included demographic variables of interest (i.e., age, race, and ethnicity) and body mass index (kg/m2), which were extracted from the electronic medical record at the time of the visit.

Statistical analyses

Frequencies, means, and standard deviations (SD) were generated for all variables. Key assumptions of multiple linear regression were tested prior to running multivariable models, including the identification of a linear relationship between the outcome variable and independent variables and no multicollinearity between independent variables. Tests of normality (Shapiro-Wilk) were conducted to examine the normal distribution of continuous variables for regression analyses. Extreme outliers were weighted prior to analysis.

Initially, two multivariable models were used to examine non-respiratory and respiratory variables in association with daytime sleepiness. Separate models were run due to the sample size limitations of including all variables in one model. The independent variables significantly associated with daytime sleepiness (at p > 0.20) were added into one model and re-run until the most parsimonious model was achieved, controlling for demographics (age, race, sex). All analyses were conducted using IBM SPSS Statistics for Windows, Version 28.0 (Released 2021; IBM Corp., Armonk, New York, United States), with statistical significance set at p < 0.05 and marginal significance set at p = 0.05.

## Results

One hundred pediatric patients aged 8 to 17 years (mean = 12.1, SD = 2.6) with a primary diagnosis of asthma were included in this study. Participants were 54% male and 45% Black with a mean number of prescribed asthma medications of 4.1 (SD = 2.2) and a mean forced expiratory volume in the first second of expiration of 97.5% (SD = 17.8).

Scores on patient surveys were mean = 14.13, range = 0-31, and SD = 6.7 for the Pediatric Daytime Sleepiness Scale; mean = 77.38, range = 15.6-100, and SD = 18.6 for the PEDS-QL; and mean = 47.2, range = 8-35, and SD = 7.2 for PROMIS. Table 1 provides a full listing of all patient characteristics.

**Table 1:** Characteristics of participants (N=100) FEV1: forced expiratory volume; BMI: body mass index

Socio-demographic characteristics	
Age (M,SD)	12.13 (2.6)
Sex (n,%)	
Male	54.0 (54)
Female	46.0 (46)
Race (n,%)	
White	45.0 (45)
Black	45.0 (45)
Other	10.0 (10)
Environmental tobacco smoke (n,%)	20 (20.2)
Respiratory-related health factors	
Severity of asthma (n,%)	
Intermittent	13.0 (13)
Mild persistent	56.0 (56)
Moderate persistent	24.0 (24)
Severe persistent	7.0 (7)
Number of asthma medications (M,SD)	4.1 (2.2)
Forced expiratory volume (FEV_1_) (M,SD)	97.5 (17.8)
Obesity (BMI > 95^th^%for age) (n,%)	36.0 (36)
In past 7 days, did you wake up at night to complete respiratory treatments? (answer=yes) (n,%)	29.0 (29)
Non-respiratory factors	
Daytime sleepiness (M,SD)	14.1 (6.7)
Health-related quality of life (M,SD)	77.4 (18.6)
Anxiety (M,SD)	47.2 (7.2)
In past 7 days, did you use cell phone one hour prior to bed? (answer=yes) (n,%)	75.0 (75)

The final regression model showed that lower patient-reported quality of life (b = -0.328, p = 0.004) and increased bedtime cellphone use (b = 0.300, p = 0.004) were significantly associated with daytime sleepiness in children with asthma. The regression model showed that increased anxiety symptoms (b = 0.213, p = 0.05), an increased number of prescribed asthma medications (b = 0.173, p = 0.05), and worse lung function (b = -0.173, p = 0.05) were marginally associated with daytime sleepiness. See Table 2 for the regression analysis.

**Table 2:** Linear regression model of daytime sleepiness (N=100)

Variable	B (SE)	Beta coefficients	95% CI	p-value
Age	-.061 (.256)	-.024	[-.570, .488]	.812
Sex	.055 (1.216)	.004	[-2.366, 2.477]	.964
Race	.333 (.893)	.033	[-1.445, 2.111]	.710
# of prescribed medications	.524 (.264)	.173	[-.001, 1.050]	.050
Quality of life	-.118 (.039)	-.328	[-.197, -.039]	.004
Anxiety	.198 (.100)	.213	[-.001, .398]	.051
Bedtime cellphone use	4.593 (1.551)	.300	[1.506, 7.681]	.004
Forced expiratory volume	-.065 (.032)	-.173	[-.129, .000]	.049

## Discussion

Daytime sleepiness is a common challenge in YWA and contributes to diminished function in this group. To develop effective interventions and treatment strategies to address this concern, it is important to understand the key variables that relate to daytime sleepiness in this population.

A key and novel finding of this study is that more patient-reported non-respiratory factors, such as bedtime cellphone usage, lower health-related quality of life, and increased anxiety, were related to daytime sleepiness compared with respiratory factors. These findings suggest that although respiratory variables contribute to daytime sleepiness in this population, non-respiratory factors may also be important clinical targets to mitigate daytime sleepiness. Strategies to improve bedtime technology usage, address anxiety-related concerns, and improve quality of life may be important components of interventions targeting daytime sleepiness.

In addition to improving daytime sleepiness and daytime function, addressing impaired sleep health may improve important medical outcomes in this group, including asthma control. For example, among adolescents and adults with asthma, Meltzer et al. demonstrated that short sleep duration is associated with reduced lung function, increased asthma attacks, and more frequent use of medication [21]. Although emerging data exists indicating that optimizing sleep health can improve asthma control, current international asthma guidelines do not address non-respiratory factors related to sleep health, indicating a novel treatment area exists in pediatric asthma management [22].

The results of this study must be considered in conjunction with its limitations. The cross-sectional design prohibits the determination of causality or the direction of effect. Indeed, many of the explanations explored may be bidirectional. Some measurement limitations also exist, including the self-report nature of the surveys and the lack of validation of the investigator-designed survey assessing technology use and respiratory symptoms. Future work using validated objective measurements and including additional sleep health domains is necessary to confirm these results. Finally, a limitation of this study is the overall relative health of the participants at the time of study visits (during periods of asthma control). Although the study included individuals with lower lung function and severe asthma, most participants had mild asthma, and their mean lung function fell within the normal range, representing a group of patients with well-controlled asthma. Furthermore, this study assessed children during routine clinic appointments; therefore, the results may not represent daytime sleepiness during asthma flares when both respiratory symptoms and other non-respiratory factors (quality of life, anxiety) may be exacerbated compared with baseline.

## Conclusions

Management of daytime sleepiness as an aspect of pediatric sleep health in YWA has implications for both daytime function and asthma control. In addition to addressing sleep-related airway concerns and managing nocturnal asthma symptoms, this study demonstrated that it is important for clinicians to address non-respiratory factors that affect sleep. Longitudinal and interventional studies are needed in this group to assess underlying mechanisms and behavioral modifications that may improve sleep health among YWA.

## Appendices

**Table 3:** Investigator-designed survey questions Answer choices: never, 1-2 nights/week, 3-6 nights/week, or every night

How often, in the prior 7 days, did you…
Use your cell phone within 1 hour of bedtime?
Wake up at night to complete respiratory treatments?
Wake up at night due to cough?
Wake up at night due to wheeze?
